# Co-creating Virtual Reality Interventions for Alcohol Prevention: Living Lab vs. Co-design

**DOI:** 10.3389/fpubh.2021.634102

**Published:** 2021-03-15

**Authors:** Timo Dietrich, Julie Dalgaard Guldager, Patricia Lyk, Lotte Vallentin-Holbech, Sharyn Rundle-Thiele, Gunver Majgaard, Christiane Stock

**Affiliations:** ^1^Griffith Business School, Social Marketing @ Griffith, Griffith University, Brisbane, QLD, Australia; ^2^Centre for Youth Substance Abuse Research, Faculty of Health and Behavioural Science, University of Queensland, Brisbane, QLD, Australia; ^3^Unit for Health Promotion Research, Department of Public Health, University of Southern Denmark, Esbjerg, Denmark; ^4^Research Department, University College South Denmark, Haderslev, Denmark; ^5^Game Development and Learning Technology, The Maersk Mc-Kinney Moller Institute, University of Southern Denmark, Odense, Denmark; ^6^Centre for Alcohol and Drug Research, Department of Psychology and Behavioural Sciences, Aarhus University, Aarhus, Denmark; ^7^Charité - Universitätsmedizin Berlin, Corporate Member of Freie Universität Berlin, Humboldt-Universität zu Berlin, and Berlin Institute of Health, Institute for Health and Nursing Science, Berlin, Germany

**Keywords:** co-creation, co-design, Living Lab, virtual reality, prevention, alcohol, adolescents

## Abstract

Addressing the need for collaborative involvement in health intervention design requires application of processes that researchers and practitioners can apply confidently to actively involve end-users and wider stakeholder groups. Co-creation enables participation by focusing on empowering a range of stakeholders with opportunities to influence the final intervention design. While collaboration with users and stakeholders during intervention design processes are considered vital, clear articulation of procedures and considerations for various co-creation methodologies warrants further research attention. This paper is based on two case studies conducted in Australia and Denmark where researchers co-created virtual reality interventions in an alcohol prevention context. This paper explored and reflected on two co-creation methods–co-design and the Living Lab—and showcased the different processes and procedures of each approach. The study demonstrates that both approaches have merit, yet highlights tensions in distinguishing between the application of each of the respective steps undertaken in each of the processes. While a lot of similarities exist between approaches, differences are evident. Overall, it can be said that the Living Lab is broader in scope and processes applied within the Living Labs approach are more abstract. The co-design process that we applied in the first case study is described more granularly delivering a clear a step-by-step guide that practitioners can implement to co-design solutions that end-users value and that stakeholders support. An agenda to guide future research is outlined challenging researchers to identify the most effective co-creation approach.

## Introduction

Collaboration with users and stakeholders during intervention design is recommended, but clear documentation of the procedures and considerations for different co-creation methodologies have only recently emerged (1–4) with the need for more work to guide practice and understand relative effectiveness of different co-creation approaches noted. Co-creation ensures that programs are designed with those that are ultimately the recipients of a health intervention. Co-creation is an umbrella term that gained popularity in the early 2000s emerging in areas including (but not limited to) management (5) and software design (6). Co-creation literature focuses on centering service solution development on users, originating from participatory design work dating back to the early 70s (6). Numerous methods have emerged over time and include design thinking (2), co-design (7), co-production (8), and Living Labs (9) highlighting a range of different approaches that can be implemented for intervention co-creation. This paper highlights and contrasts two popular co-creation approaches, namely co-design and the Living Lab.

Co-design is a scientific method of data collection with the aim of including consumers affected by a health intervention (4). Co-designed programs have demonstrated effectiveness across health (10) and environmental issues (1) and thus demonstrated value for researchers, users and society at large (11). More recently, the need to include wider stakeholder groups during the intervention design process has been identified (4) and processes seeking to involve stakeholders within the co-design process to ensure that user solutions identified are feasible have emerged (12). Co-design processes ensure that emphasis is placed on empowering participants and that all solutions emerging from co-design are user centered and stakeholder supported. Recent co-design process models (4, 12) have begun to articulate the necessity to think beyond ideation and gauge how user generated ideas can be translated into effective intervention programs that are endorsed by end-users and stakeholder groups.

The Living Lab methodology is defined as “*a design research methodology aimed at co-creating innovation through the involvement of aware users in a real-life setting”* [(9), p. 139]. Living Labs have been applied in urban settings (13), entrepreneurial settings (14), professional development (15), and dementia interventions (16), but all take very different structures and forms. Publications on Living Labs began to emerge in the early 2000s and have been predominantly set up and reported within the European context (17). The existing literature has positioned Living Labs as a design method that aims to achieve innovation by setting up environments that allow for end-users to experience and contribute to the solution throughout the developmental stages (9). In other words, it provides a unique setting for collective innovation involving heterogeneous stakeholders such as but not limited to citizens, customers, policy makers, researchers, educators, businesses and universities (18, 19). Living labs remain however significantly underexplored in the academic literature and require further empirical exploration to demonstrate more clearly the scope, benefits and limitations to the approach. Schuurman et al. point out “…*[the literature] positions Living Labs too much as an “everything is possible” concept that resembles an empty box, in the sense that you can put whatever methodology or research approach inside”* [(17), p. 12].

This paper aims to provide a methodological comparison between two co-creation methods (co-design and Living Lab) to highlight key considerations as well as a comparison of both processes. This study draws its data from two virtual reality case studies, namely a co-design study conducted in Australia and a Living Lab study delivered in Denmark where researchers co-created virtual reality (VR) interventions in an alcohol prevention context. Both virtual reality interventions consist of the simulation of a party situation in which the user can experiment with different communication and behavioral options and both virtual reality interventions are aimed at strengthening alcohol resistance skills. The method section provides the contextual background as well details around how each method was applied to co-create the virtual reality interventions. Next, the paper summarizes the findings and critically discusses and contrasts both co-creation processes.

## Method

### Case Description of the Co-creation Cases

#### Blurred Minds VR House Party

The first case study describes and summarizes the research team's experience with the co-design process to co-create the Blurred Minds VR House Party (20, 21). The seven-step co-design process of (1) resourcing, (2) planning, (3) recruiting, (4) sensitizing, (5) facilitating, (6) reflecting, and (7) building for change (see Table 1 below) was used (4). Process and outcomes evaluations for the Blurred Minds House Party are reported in Dietrich et al. (20). This sequential step-by-step process was developed to guide the discovery of new, innovative intervention ideas (4).

**Table 1:** Co-designing the Blurred Minds VR House Party.

**Co-design process**	**Application to Blurred Minds VR house party**
Resourcing	A systematic literature review investigated the application of VR to alcohol education interventions (22). Additional resources were reviewed to gain further insights into VR's latest technological developments to ensure that solution development would reflect the latest technological VR standards. The team also consulted with two film and one VR expert throughout the development process.
Planning	A multi-disciplinary team of researchers with expertise in social marketing, gamification, and service design planned the co-design sessions. The team prepared components of the co-design session, such as the development of a run-sheet, screening survey, group activities, and design tools (21).
Recruitment	Leveraging existing school network contacts, the team recruited a group of students from a public secondary school. Close collaboration with the teacher of the school ensured that we were able to set expectations for the sensitization and co-design workshop phases as well as secure a location on school grounds to run the workshop [more details available in (21)].
Sensitizing	Adolescents had the opportunity to test pilot versions of four newly developed online games for Blurred Minds. This sensitizing phase provided students with a relevant and fun way to engage with the notion of alcohol education resources prior to taking part in the co-design session.
Facilitation	The session commenced with screening survey, a brief introduction of the research team as well as highlighting the aims of the workshop. Next, the team showed an interactive simulation experience and a head-mounted VR display to showcase what type of virtual reality experience the team was aiming to create. Four teams were formed by the researchers and teams were provided with tools to help them co-create a virtual house party that would appeal to them. They were also encouraged to role play, experience interactive videos and wear beer goggles to help them understand the purpose and aim of the session in a playful manner. Design tools in form of butcher paper, stickers, markers, coloring pens and post-it notes were distributed. The workshop finished with short presentations of each student team showcasing their work to the entire group [more details available in (21)].
Reflecting	All data derived from the developed ideas as well as the presentation transcripts were coded and thematically analyzed. These user insights were taken into consideration when producing the final version of the Virtual House Party scripts and when planning production details. Co-design in this case provided important insights into ensuring that both the language used and the party setting depicted were realistic for the young audience. The final scripts and party planning were created with professional script writers and film producers.
Building for Change	The team consulted a wide stakeholder group prior to production of the VR experience including alcohol and drug experts, a VR expert, and two experienced film producers with an interest in interactive storytelling using VR. The VR simulation was developed and focused on strengthening self-efficacy and changing attitudes toward excessive drinking (20). The script was written around key theoretical outcome measures (underpinned by Social Cognitive Theory) and was filmed using a 360° video camera to provide users with an experience that would resemble a real-life scenario. More details on the VR intervention development and Blurred Minds as well as preliminary effectiveness findings are published in (20).

#### VR FestLab

The second case study describes and summarizes the research team's experience with the Living Lab method. The Living Lab method was applied to co-create a gamified (VR) simulation—VR FestLab (25). User experiences for VR FestLab are reported in Guldager et al. (24).

This project used the Living Lab process which was comprised of six individual steps namely (1) exploration of key concepts, (2) concept design, (3) prototype design, (4) innovation design, (5) testing the product, and (6) evaluation of the process and the product (26) (Table 2).

**Table 2:** Using the Living Lab method to co-create VR FestLab.

**Living Lab process**	**Application to VR FestLab**
Exploration of key concepts	The existing “VR House Party” film script from the Australian Blurred Minds alcohol education program (21) was revised by the development group, consisting of two prevention practitioners, two prevention scientists, two social marketing scientists, two VR game scientists, one VR game designer, one film production expert and eleven students from a folk high school who represented young end-users. Further, the researcher and VR tool developer from Blurred Minds shared his knowledge and lessons learned from the development, delivery and evaluation of Blurred Minds with the group. The group explored the existing Blurred Minds VR game and reflected on their experiences hereof. Finally, the development group co-created a list of elements which should be maintained, changed or added in order to make the prototype of the Danish VR simulation fit contextually and culturally to a Danish party setting.
Concept design	Based on the output from the exploration stage and facilitated by the film production and VR game design expert, the students co-created a film script for the gamified VR simulation. The film script was presented to the development group through role-play and flow-charts of the storyline and a list of improvements and changes was created. This stage resulted in a film-manuscript which included a comprehensive storyboard and descriptions of the characters to be casted.
Prototype design	The students from the development group produced the 360-degree videos for the VR simulation in collaboration with the film production expert and the game design researcher. The students were responsible for casting and directing the boarding school students (aged 15-17 years) who served as actors. The videos were optimized with the support of a professional film editor. Next, the videos were combined in a game engine platform and interactivity elements were added, resulting in version 1 of the digital prototype. More details on the development of the tool are published in (23).
Innovation design	Version 1 of the digital prototype was presented to the development group by the two VR game scientists. At this stage, the film students were not represented in the development group, because they had graduated from the school. The remaining group (prevention practitioners, prevention scientists, social marketing scientists, VR game scientists, VR film production expert) examined and explored the prototype and shared their experiences and feedback about the prototype. This stage resulted in a co-developed list of priorities for improvement. The digital prototype was improved accordingly (version 2). Additional graphical elements were added to improve the user experience and to guide the user.
Testing the product	The improved prototype (version 2) was tested with 31 boarding school students (average age 16 years) focusing on usability, technical qualities and user satisfaction and general feedback. A list of issues resulted from this and minor improvements were made for version 3 of the digital prototype. More details on the results of the pilot testing are described in (24).
Evaluation of the process and product	To evaluate the co-creation process, the development group shared their experiences of developing and pilot testing the VR game at a meeting. An outcome of this was a co-created list of lessons learned.

## Results and Discussion

This study was informed by two case studies conducted in Australia and Denmark where researchers co-created virtual reality interventions within an alcohol prevention context. This paper explored and reflected on two co-creation methods–co-design and the Living Lab—and showcased the different processes and procedures of each approach. This paper makes two important contributions. First, it provides a methodological comparison of two different co-creation approaches that were used to design VR interventions. Second, it demonstrates that both approaches have merit, yet highlights tensions in distinguishing between the application of each of the respective steps undertaken in each of the processes. While a lot of similarities exist between approaches, differences are evident. Overall, it can be said that the Living Lab is broader in scope and processes applied within the Living Labs approach is more abstract. The co-design process that we applied in the first case study are described more granularly delivering a clear a step-by-step guide that practitioners can implement to co-design solutions that end-users value and that stakeholders support. Both approaches were able to be utilized to develop innovative VR interventions that have demonstrated initial successes (20, 23–25). Next, we discuss consideration for each of the processes from two major perspectives namely the fuzzy front end of both processes followed by reflection on the divergence and convergence of both approaches.

### From Fuzzy to Clear: The Starting Point of the Co-design and Living Lab Processes

During the co-design process, the *resourcing* stage marks an important step that informs all subsequent co-design stages of the co-design process. It provides researchers with the opportunity to collaborate closely with key stakeholders to ensure that all voices are heard prior to embarking on the subsequent six steps of the process. This can take many shapes and forms (e.g., expert interviews, literature reviews, surveys) and in this case featured a systematic literature review to understand the current state of knowledge of VR in alcohol education (22). While this information delivered a peer review evidence-based understanding of VR interventions for young people, data was outdated and it did not deliver understanding of the current possibilities that VR afforded. For this, a film and VR expert were recruited into the team to help set more realistic goals for the overall project and for the respective co-design session with students. This stage was very useful to provide the necessary clarity to inform subsequent co-design process stages. *Planning*, then operationalised the goals set in the *resourcing* phase by ensuring that all aspects of *recruitment, sensitization, facilitation*, and *reflection* were organized. The process for *resourcing, planning* and even *recruitment* has been described as highly iterative in Trischler et al. (4). The Living Lab processes suggests that a broader planning stage takes place at commencement which takes into consideration diverse stakeholder views and stresses the importance of creating value for the user and discussing when in the process users can contribute (26). The Living Lab process used in the second case study featured six phases and commenced with the *Exploration of key concepts* where a big focus was placed on the aim and scope of the virtual intervention build. It was important to gain information on the previous research project Blurred Minds to understand best practices as well as lessons learned to most cost-effectively create VR FestLab. A wide range of stakeholders were consulted and tasked with identifying who the end-users are, what important characteristics they share, and where users could contribute throughout the Living Lab process (26). In summary, both approaches aimed to pinpoint a clear aim of the project, and both processes identified expert stakeholders to inform the subsequent user focused process.

### Divergence and Convergence of Both Approaches

While the seven-step co-design process focusses on preparing for the specific co-design sessions with users and stakeholders through *sensitization* and *facilitation*, the Living Lab process is more focussed on the creation of an initial *concept design*, followed by a more concrete *prototype design*, and a more finalized *innovation design*. It is important to achieve incremental improvements while carefully ensuring that user and stakeholder voices are heard throughout these key procedural steps. For example, a concept needs to be detailed enough so that end-users can understand and engage with the initial concept, while allowing room for open and constructive exploration of other concepts during end-user engagement. Concepts can take the form of storyboards, visual narratives and other mock-ups (26). It is important to note that these concepts have also been brought to co-design sessions, however these are covered in the initial stages of the co-design process (*resourcing & planning)* *(**4**)*. Next, the Living Lab process outlines a *prototype design* stage which selects the winning *concept design* from the previous step and then articulates—and potentially builds a mock-up entailing “basic functions, workflows and interfaces” [(26), p. 34]. Taken together, the Living Lab process places much greater emphasis on prototyping than co-design. For example, in the Danish context researchers ended up with three iterations of the prototype while in in co-design only one prototype was built. This is an important distinction and leaves room for co-design processes to be improved.

During the co-design process the focus is directed toward end-user and stakeholder engagement during co-design sessions. Specifically, *sensitizing* allows for participants to appropriately engage with the aims of the co-design workshops (27). This can be playful, serious or creative. We used online games to engage adolescents with relevant and fun content to spark creativity and provide them with a fun environment that would foster creativity and would help them understand what the Blurred Minds program aimed to do. *Facilitation* welcomes participants and uses warm-up activities to assist in developing trust, empowering participants to contribute and foster creativity and collaboration among team members. Sensitisation and facilitation are very specific steps ensuring user and stakeholder engagement and empowerment during co-design are evident. Interestingly, both are however not visible in the Living Lab processes discussed (26). While this marks an important divergence of both processes, it demonstrates important and very helpful information that facilitators of Living Labs would benefit from. Currently, Living Lab resources refer to interviews and observations with users to ensure that their needs are met. However, users are not necessarily viewed as experts of their own experiences but rather as a checkpoint in an innovation process.

Next, the co-design steps focus their attention on a detailed and immersive *reflection* following the co-design sessions. It is important to note that the co-design process at this point has not yet commenced a more detailed prototype development which is significantly different from the Living Lab process. *Reflecting* completes the analysis of all obtained data from the co-design workshops which often features a mix of qualitative and quantitative data (28, 29) and more recently stakeholder input to assess feasibility of user generated ideas (12). These learnings can then be derived into key insights that shape the direction of the intervention development while carefully gauging feasibility, project team capacity, and target audience wants and needs (4, 30). Finally, and only at the *building for change* step, the process asks for the development of prototypes which should be based on the insights generated through the six previous steps. It is also important to note that this is a newer and more recent addition to co-design process (4), where stakeholder input is sought to assess feasibility of user generated ideas (12). Moving forward, working closely with experts, stakeholders and end-users to build concrete prototypes through fast moving iteration cycles is recommended as an addition to existing co-design processes to ensure that prototypes get tested to compare and contrast user acceptance of generated solutions. The Living Lab process as it was applied in the Danish case concluded with *testing of one product* with end-users and with *evaluation of the product and process* with all stakeholders involved in the process, but evaluation might also be included in addition during earlier Living Lab stages (26).

### Limitations and Future Research

This study is not without limitations. First, whilst these two co-creation methods were undertaken in the same context (alcohol education for adolescents), Blurred Minds did inform the VR FestLab, meaning that the VR FestLab benefitted from the early learnings gained in the design, implementation and evaluation of Blurred Minds. This may have influenced the dynamics and outcomes achieved during the Living Lab process. However, we are not able to articulate the scale of this influence on the process. We note that many different descriptions of co-design processes and Living Lab approaches exist. It is important to contrast and distinguish the various co-creation approaches to assist researchers and practitioners seeking to understand the relative strengths and weaknesses of approaches. By comparing and contrasting approaches assumptions can be questioned and enhanced understanding can be gained. Both, Living Lab and co-design, still lack empirical research and we know relatively little about the effectiveness of various approaches. Future research should replicate this methodological comparison simultaneously and compare the processes and the outcomes, such as differences in the participants' engagement level and a final outcome evaluation to permit a full cost benefit analysis. This research agenda will enable understanding of how programs can be co-created most effectively into interventions that are capable of achieving desired outcomes.

### Conclusion

Co-creation requires bringing together a group of people that collectively design relevant and engaging health intervention solutions without a dominant single voice taking over the process. This study contrasted two case studies that aimed to co-create virtual reality interventions within an alcohol prevention context. Both, the co-design and the Living Lab method, demonstrated utility to design innovative health intervention solutions that have demonstrated initial positive successes.

## Data Availability Statement

The data analyzed in this study is subject to the following licenses/restrictions: Additional information can be made available. Requests to access these datasets should be directed to t.dietrich@griffith.edu.au.

## Author Contributions

TD wrote the first draft of the manuscript and participated in the data collection and analysis of the Australian case study. CS was responsible for and participated in the data collection of the Danish case study and contributed to the drafting of the manuscript. JG, LV-H, and PL participated in the data collection for the Danish pilot study. TD and CS were principle investigators of the respective studies, had the lead in its conception and coordination. All authors contributed to the manuscript, critically reviewed its content, and have read and agree to the published version of the manuscript.

## Conflict of Interest

The authors declare that the research was conducted in the absence of any commercial or financial relationships that could be construed as a potential conflict of interest.
